# Therapeutic potential of deuterium‐stabilized (*R*)‐pioglitazone—PXL065—for X‐linked adrenoleukodystrophy

**DOI:** 10.1002/jimd.12510

**Published:** 2022-05-19

**Authors:** Pierre‐Axel Monternier, Jaspreet Singh, Parveen Parasar, Pierre Theurey, Sheila DeWitt, Vincent Jacques, Eric Klett, Navtej Kaur, Tavarekere N. Nagaraja, David E. Moller, Sophie Hallakou‐Bozec

**Affiliations:** ^1^ Poxel SA Lyon France; ^2^ Department of Neurology Henry Ford Health System Detroit Michigan USA; ^3^ DeuteRx, LLC Andover Massachusetts USA; ^4^ Department of Medicine, Division of Endocrinology University of North Carolina School of Medicine Chapel Hill North Carolina USA; ^5^ Department of Neurosurgery Henry Ford Health System Detroit Michigan USA

## Abstract

X‐linked adrenoleukodystrophy (ALD) results from ABCD1 gene mutations which impair Very Long Chain Fatty Acids (VLCFA; C26:0 and C24:0) peroxisomal import and β‐oxidation, leading to accumulation in plasma and tissues. Excess VLCFA drives impaired cellular functions (e.g. disrupted mitochondrial function), inflammation, and neurodegeneration. Major disease phenotypes include: adrenomyeloneuropathy (AMN), progressive spinal cord axonal degeneration, and cerebral ALD (C‐ALD), inflammatory white matter demyelination and degeneration. No pharmacological treatment is available to‐date for ALD. Pioglitazone, an anti‐diabetic thiazolidinedione, exerts potential benefits in ALD models. Its mechanisms are genomic (PPARγ agonism) and nongenomic (mitochondrial pyruvate carrier—MPC, long‐chain acyl‐CoA synthetase 4—ACSL4, inhibition). However, its use is limited by PPARγ‐driven side effects (e.g. weight gain, edema). PXL065 is a clinical‐stage deuterium‐stabilized (*R*)‐enantiomer of pioglitazone which lacks PPARγ agonism but retains MPC activity. Here, we show that incubation of ALD patient‐derived cells (both AMN and C‐ALD) and glial cells from Abcd1‐null mice with PXL065 resulted in: normalization of elevated VLCFA, improved mitochondrial function, and attenuated indices of inflammation. Compensatory peroxisomal transporter gene expression was also induced. Additionally, chronic treatment of Abcd1‐null mice lowered VLCFA in plasma, brain and spinal cord and improved both neural histology (sciatic nerve) and neurobehavioral test performance. Several in vivo effects of PXL065 exceeded those achieved with pioglitazone. PXL065 was confirmed to lack PPARγ agonism but retained ACSL4 activity of pioglitazone. PXL065 has novel actions and mechanisms and exhibits a range of potential benefits in ALD models; further testing of this molecule in ALD patients is warranted.

## Graphical abstract

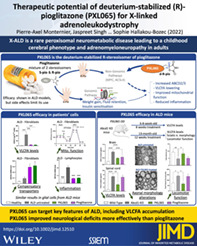

## 1 INTRODUCTION

X‐linked adrenoleukodystrophy (ALD) is a rare neurometabolic disease caused by mutations in the ATP Binding Cassette subfamily D, member 1 (ABCD1) gene. These mutations lead to impaired function of the adrenoleukodystrophy Protein (ALDP), preventing the transport of Very Long Chain Fatty Acids (VLCFA) into peroxisomes.^1^ As VLCFA cannot enter the peroxisomes to be oxidized, they accumulate in plasma^2^ and tissues.^1^ Thus, increased circulating and tissue levels of VLCFA, in particular saturated C26 (C26:0) and C24 (C24:0), have been described as the hallmark of the pathology. Importantly, elevated cellular C26:0 levels are established as the proximate cause of disease^3^, ^4^ and are believed to be responsible for oxidative stress, inflammation, and mitochondrial dysfunction, leading to neuronal atrophy and demyelination.^5^, ^6^, ^7^

There are two main clinical subtypes of ALD. Adrenomyeloneuropathy (AMN) is an adult, slowly progressive disease leading to severe disabilities. Its pathophysiology is characterized by spinal cord and peripheral nerve degeneration that lead to spastic paraparesis and sensory ataxia.^1^ The second main form, cerebral ALD (C‐ALD), typically occurs during childhood (but also in adults) and is characterized by brain white matter lesions and the rapid progression of neurologic deficits and death, caused by a rapid inflammatory demyelination process.^8^

Although allogeneic hematopoietic stem cell transplantation is now employed to treat early‐stage C‐ALD, there is currently no approved pharmacologic treatment available for AMN or C‐ALD. Hence, it is critically important to investigate potential therapeutics that could either halt the progression of AMN or treat and prevent the onset of C‐ALD.

Pioglitazone—a marketed antidiabetic thiazolidinedione (TZD) drug—improved the disease phenotype of Abcd1‐null mice by normalizing bioenergetic parameters and decreasing oxidative stress in spinal cord and ameliorating impaired locomotor function.^9^ Additionally, leriglitazone, a related TZD (the M‐IV metabolite of pioglitazone), which is currently under development for ALD, also exhibited an encouraging preclinical profile suggesting that this class of compounds is of high interest to treat ALD.^10^ The efficacy observed to‐date with both pioglitazone and leriglitazone has been attributed to transcriptional actions as agonists of peroxisome proliferator activated receptor γ (PPARγ).^9^, ^10^

Pioglitazone is a racemic mixture of interconverting (*R*)‐ and (*S*)‐enantiomers. We recently discovered that only the (*S*)‐enantiomer exerts potent PPARγ activity, whereas both the (*R*)‐ and (*S*)‐enantiomers (and the parent racemate) have other—nongenomic—actions.^11^ PXL065 is a stabilized form of (*R*)‐pioglitazone created by the addition of deuterium to its chiral center.^11^ In cell culture and in vivo, PXL065 retains pioglitazone's nongenomic activity with substantially less PPARγ agonism, the latter leading to undesired TZD side effects (body weight gain and edema).

Here, we investigated the effects of PXL065 on hallmarks of ALD in both in vitro and in vivo models. In diseased patient‐derived cells, multiple beneficial effects on cellular phenotypes, including correction of elevated VLCFA, were documented. In addition, chronic administration of PXL065 to Abcd1‐null mice produced distinct reductions in plasma and tissue VLCFA accumulation along with improvements in other disease‐associated phenotypes. As PXL065 has recently completed Phase I trials in healthy subjects, the clinical trial assessment of its potential effects in patients with ALD (adult male AMN) is planned to initiate in 2022.

## 2 MATERIAL AND METHODS

### 2.1 In vitro studies

#### 2.1.1 Work system

##### Compounds

PXL065 was synthetized by AAPharmaSyn; pioglitazone was purchased from Dr. Reddy's Laboratories (India); compounds were dissolved in DMSO.

##### Human‐derived cells

Primary human fibroblasts (healthy: GM03348, AMN: GM17819, C‐ALD: GM04934), and immortalized human lymphocytes (healthy: GM03798, C‐ALD: GM04673) were obtained from the NIGMS Human Genetic Cell Repository, Coriell Institute for Medical Research (www.coriell.org) and cultured as previously described.^12^

##### Mouse‐derived glial cells

Mouse primary mixed glial cells were prepared from 2‐day‐old wild‐type (WT) and Abcd1 null pups, as previously described.^13^

#### 2.1.2 mRNA levels

0.5 million cells/well were plated in 6‐well plates. After 72 h of compound exposure (cells at 80% confluence), mRNA was isolated (Qiagen RNAeasy kit, #74104) and cDNA was prepared (Qiagen RT II cDNA kit [Qiagen, #330401]). Real‐time quantitative polymerase chain reaction (PCR) was conducted using Bio‐Rad CFX96 Real‐Time PCR Detection System and iTaq™ Universal SYBR® Green Supermix (Bio‐Rad, #1725124). Results are expressed as ratio of the targeted gene over the housekeeping gene, RLP27.

#### 2.1.3 VLCFA content

300 K cells were plated in 100‐mm petri plates. Cells were treated for 7 days. At the end of treatment (cells at 80% confluence), cells were washed with ice‐cold phosphate buffered saline and 1 million cells per sample were pelleted and provided to the Lipidomic Core for VLCFA analysis as described in Appendix S1.

#### 2.1.4 Bioenergetics

0.5 million cells/well were plated in 6‐well plates. Cells were treated for 72 h (cells at 80% confluence at the end of treatment). They were then harvested with cell lifters and washed twice with bicarbonate‐free DMEM prewarmed at 37°C. 1 million cells/well were plated in 175 μl of bicarbonate‐free DMEM in XFe96 plates and preincubated at 37°C for 1 h for degassing. Oxygen consumption rate (OCR) was measured using Seahorse XFe96 Analyzer (Agilent) as previously described.^12^

#### 2.1.5 Long‐chain acyl‐CoA synthetase (ACS) activity

ACSL enzyme activity assays were performed using purified recombinant rat FLAG‐ACSL1 and FLAG‐ACSL4 with [^14^C] palmitate and a scintillation counter as described in Appendix S1.

### 2.2 PPARγ agonist assay

PPARγ agonist activity was measured using histidine‐tagged‐PPARγ, biotin‐tagged‐TRAP220 coactivator and fluorescence acceptor/donor as described in Appendix S1. The results are expressed as a percent of the control response to 10 μM rosiglitazone.

### 2.3 Inflammatory gene expression profiling

C‐ALD patient‐derived lymphocytes were cultured in Serum‐free DMEM for cytokine mRNA analysis for 72 h. Abcd1‐null glial cells were cultured in serum‐free media overnight followed by 2 h pretreatment with tested compounds; TNFα (10 ng/ml) and IL1β (10 ng/ml) were then added to the media for 70 h resulting in a total compound exposure period of 72 hrs. mRNA levels were measured as described above.

### 2.4 In vivo experiments

#### 2.4.1 Animals

Animals were handled following the appropriate ethical guidelines as described in Appendix S1. All institutional and national guidelines for the care and use of laboratory animals were followed. Genotyping was performed by PCR as described in Appendix S1.

### 2.5 VLCFA assessment in 6‐ to 8‐week‐old and 13‐month‐old Abcd1‐null mice

6‐ to 8‐week‐old Abcd1‐null male mice were treated with PXL065, pioglitazone (15 mg/kg), or methylcellulose vehicle once daily (QD, oral gavage) for 8 weeks. VLCFA content was measured in plasma, spinal cord, and brain after 8 weeks of treatment. VLCFA content in spinal cord was also assessed in 13‐month‐old mice treated for 12 weeks with PXL065 or pioglitazone at 15 mg/kg QD. On the last treatment day, mice were sacrificed with CO_2_; plasma, brain and spinal cord were then harvested, snap‐frozen in liquid nitrogen and stored at −80°C until processed for VLCFA analysis (see above).

### 2.6 Behavioral tests in 13‐month‐old Abcd1‐null mice (open‐field test)

13‐month‐old Abcd1‐null male mice were treated with compound or vehicle as noted above.

Measures of locomotion and exploration, including total distance, freezing time, and number of rearings, were scored. Data were obtained as described in Appendix S1.

### 2.7 Axonal morphology

Electronic microscopy (EM) morphometric studies were performed on thin sections (1 μM) of sciatic nerve from WT and Abcd1‐null mice, as described in Appendix S1. Criteria for blinded evaluations were appearance of axons, scored as regular and nearly circular bundles or stellate (star‐like).

### 2.8 Statistics

Data were analyzed using Graph Pad Prism® V9.3.1 software. Analysis was conducted using raw values including all groups. Model characterization and significance of treatment effects were tested using one‐way analysis of variance followed by Dunnett's multiple comparisons test when data were normally distributed, and Kruskal–Wallis followed by Dunn's multiple comparisons test when data were not normally distributed.

## 3 RESULTS

### 3.1 PXL065 suppresses elevated VLCFA in patient‐derived fibroblasts

**FIGURE 1:**
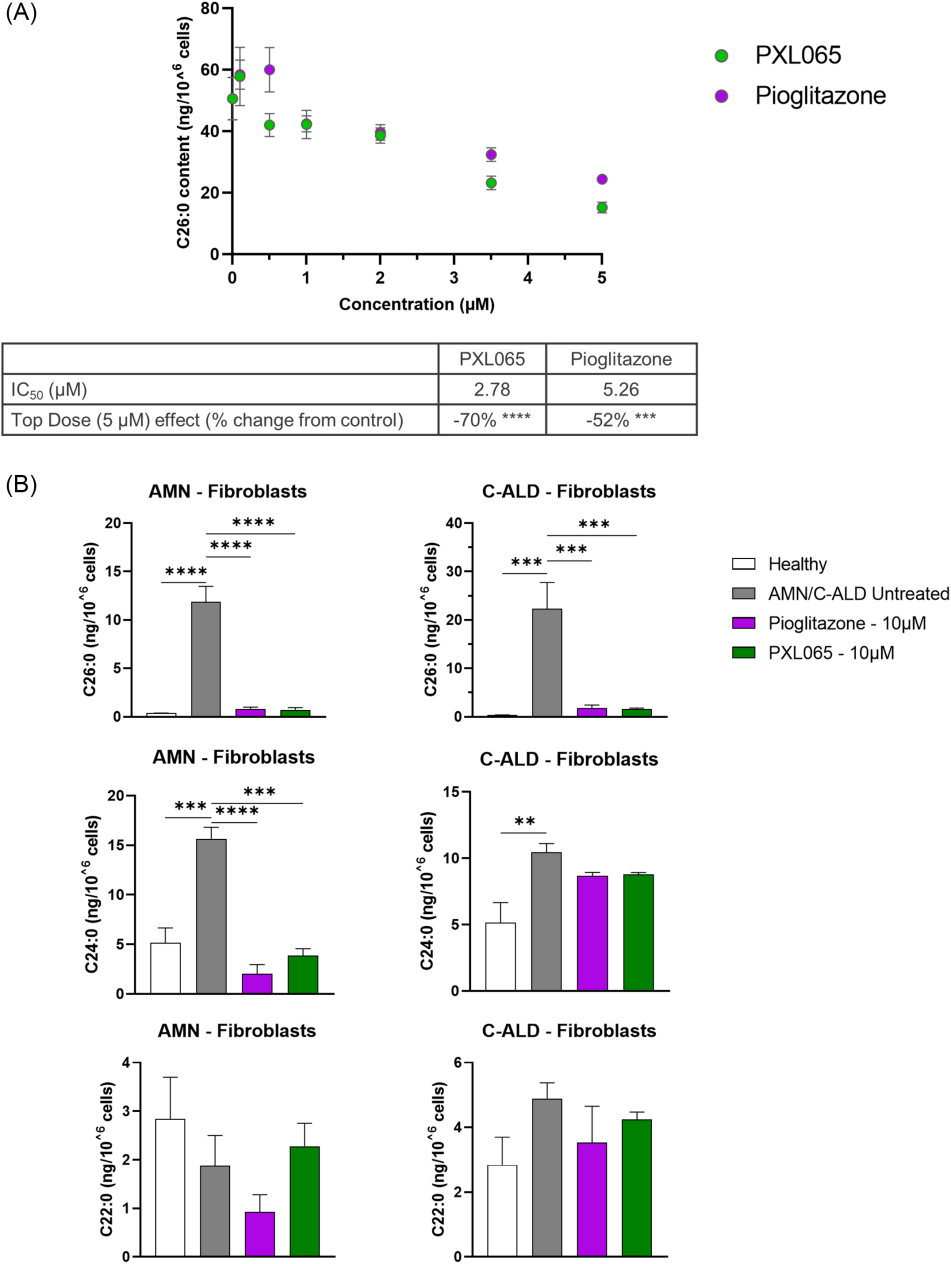
PXL065 and Pioglitazone suppress very long chain fatty acids (VLCFA) levels in fibroblasts derived from two adrenoleukodystrophy (ALD)—C‐ALD and AMN—patients. (A) Dose–response effect of PXL065, pioglitazone on C26:0 levels measured by mass spectrometry in AMN fibroblasts following incubation with compounds at 0, 0.1, 0.5, 1, 2, 3.5, and 5 μM for 7 days. IC50s were calculated using nonlinear regression analysis. Results are mean ± SEM, *n* = 5 replicates. (B) Effect of PXL065 and pioglitazone on VLCFA levels measured by mass spectrometry in AMN and C‐ALD fibroblasts, following incubation at 10 μM for 7 days. Results are mean ± SEM, *n* = 3 replicates/condition/patient. Beneficial effects of the drugs on VLCFA levels were reproduced in fibroblasts derived from these donors in two other independent experiments. ** p < 0.01, *** p < 0.001, **** p < 0.0001 by One‐way analysis of variance (ANOVA) followed by Dunnett's multiple comparison versus untreated AMN/C‐ALD cells

The dose–response range of PXL065 was determined in AMN patient‐derived fibroblasts alongside effects of pioglitazone (Figure 1A). After 7 days of treatment, PXL065 reduced elevated C26:0 in a dose dependent manner (IC_50_ = 2.78 μM); pioglitazone had a similar effect with slightly lower potency (IC_50_ = 5.26 μM). Additionally, the C26:0/C22:0 ratio was significantly decreased by PXL065 and pioglitazone indicating relative selectivity on VLCFA (data not shown). Following these results, we pursued our investigations with PXL065 in two independent sets of experiments conducted with one cell line obtained from an adult ALD patient (with AMN—“AMN fibroblasts”), and another cell line from a younger ALD patient (with C‐ALD—“C‐ALD fibroblasts”).

In confirmatory experiments also aimed at assessing maximal effects, PXL065 consistently reduced elevated C26:0 and C24:0 levels in AMN fibroblasts (Figure 1B) (−94%, p < 0.0001 and −75%, p < 0.001, respectively). In C‐ALD fibroblasts, PXL065 decreased C26:0 levels (−93%, p < 0.001) but not C24:0 when compared to untreated cells (Figure 1B). C22:0 levels (not known to be disease associated) were not elevated—and were unaffected by PXL065—in either AMN or C‐ALD cells (Figure 1B). Overall, a similar profile was observed with pioglitazone. The same pattern seen in patient‐derived fibroblasts was also observed in lymphocytes treated with PXL065 and pioglitazone (Figure S1).

### 3.2 PXL065 enhances mitochondrial function and induces expression of compensatory fatty acid transporters in patient‐derived cells

**FIGURE 2:**
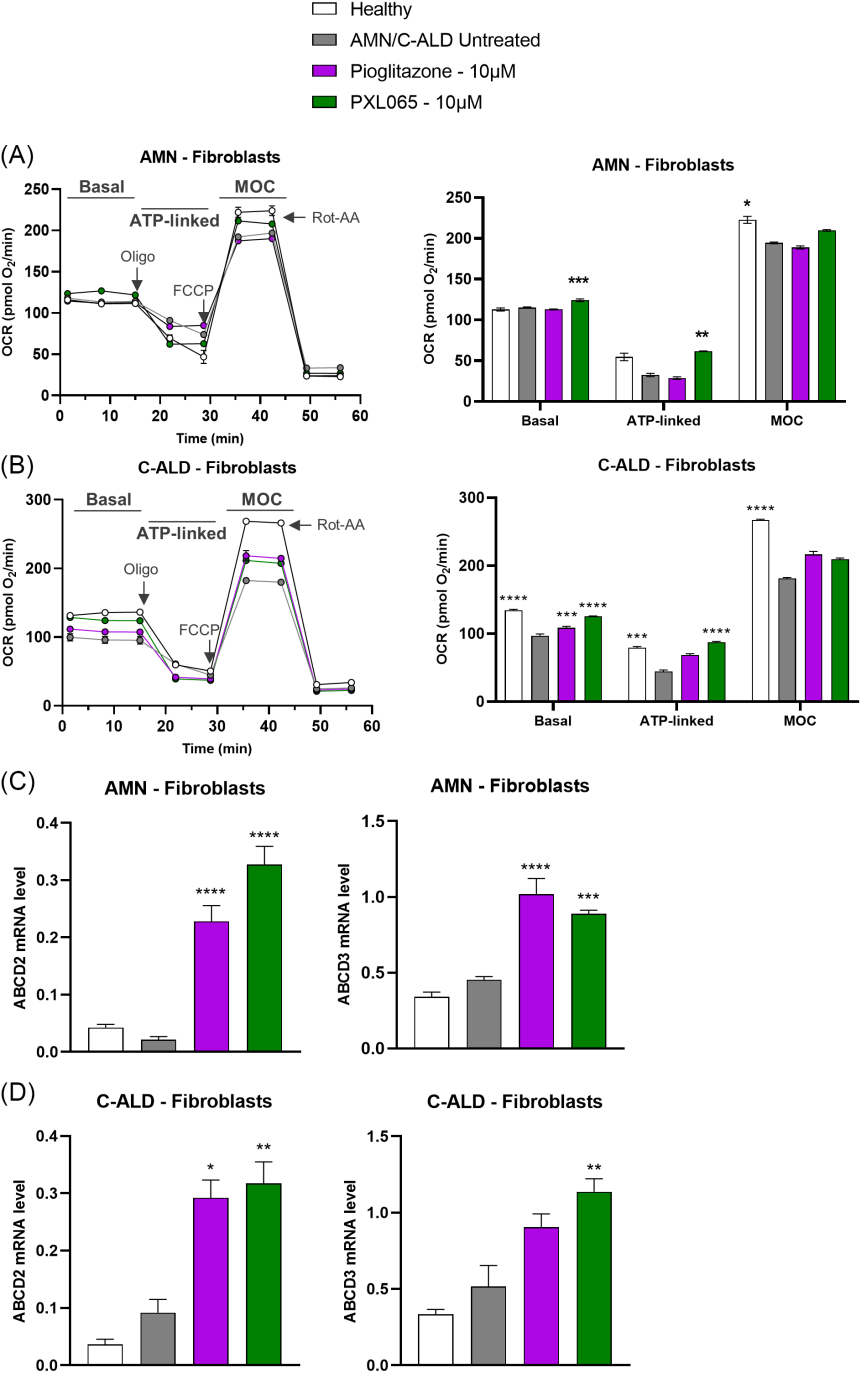
PXL065 improves mitochondrial function and increases compensatory transporters, ABCD2 and ABCD3, mRNA levels in fibroblasts derived from two ALD—C‐ALD and AMN—patients. AMN (A) and C‐ALD (B) fibroblasts were exposed for 72 h to PXL065 or Pioglitazone at 10 μM. Bioenergetics analysis was performed using a Seahorse Analyzer and parameters were evaluated by sequential additions of: oligomycin (Oligo—1 μM), FCCP (0.25 μM) and Rotenone‐Antimycin A (Rot‐AA—1 μM). Basal is first three measurements, ATP‐linked is oxygen consumption rate (OCR) drop following oligo addition, Maximal Oxidative Capacity (MOC) is OCR following addition of FCCP. Results are mean ± SEM, *n* = 6 replicates/condition/patient. Cells were exposed for 72 h to PXL065 or pioglitazone at 10 μM prior to mRNA level analysis by RT‐qPCR in AMN (C) and C‐ALD fibroblasts (D). Results are mean ± SEM, *n* = 3–6 replicates/condition/patient. * p < 0.05, ** p < 0.01, *** p < 0.001, **** p < 0.0001 by One‐way analysis of variance followed by Dunnett's multiple comparison versus untreated AMN/C‐ALD cells

As mitochondrial dysfunction is a key feature of cells from ALD patients,^14^ we further assessed this disease component. In untreated AMN fibroblasts, no clear phenotype of dysfunction was evident (Figure 2A). In contrast, in C‐ALD fibroblasts, basal and ATP‐linked OCR were reduced compared to healthy cells, by −27% (p < 0.0001) and −44% (p < 0.001), respectively (Figure 2B). PXL065 treatment of C‐ALD fibroblasts improved basal (+29%, p < 0.0001) and restored ATP‐linked OCR (dedicated to ATP synthesis, +100%, p < 0.0001). Despite the lack of phenotype, PXL065 treatment also increased basal and ATP‐linked OCR in AMN fibroblasts (+7%, p < 0.001 and + 87%, p < 0.05, respectively). Maximal Oxidative Capacity (MOC) was consistently decreased across cell lines when compared to healthy cells; however small quantitative improvements mediated by PXL065 did not reach statistical significance. Relative to PXL065, pioglitazone appeared to produce more limited effects on mitochondrial function. Similar results were observed in C‐ALD lymphocytes (Figure S2).

Transporters related to ABCD1, namely ABCD2 and ABCD3, are reportedly able to subserve as alternative means of peroxisomal VLCFA import when ABCD1 is absent or deficient.^15^ We evaluated their expression by measuring mRNA levels following treatment with PXL065. No differences were observed in untreated patient—versus healthy cells (Figure 2C,D). In AMN fibroblasts, PXL065 induced the overexpression of ABCD2 by 15‐fold (p < 0.0001), while ABCD3 increased by two‐fold (p < 0.001) (Figure 2C). In C‐ALD fibroblasts, PXL065‐induced ABCD2 mRNA by three‐fold, (p < 0.01) and ABCD3 by 2‐fold (p < 0.01) (Figure 2D). Pioglitazone exhibited a similar profile in AMN and C‐ALD fibroblasts. Similar results were observed in AMN lymphocytes (Figure S3).

### 3.3 Effects in glial cells derived from Abcd1‐null mice

**FIGURE 3:**
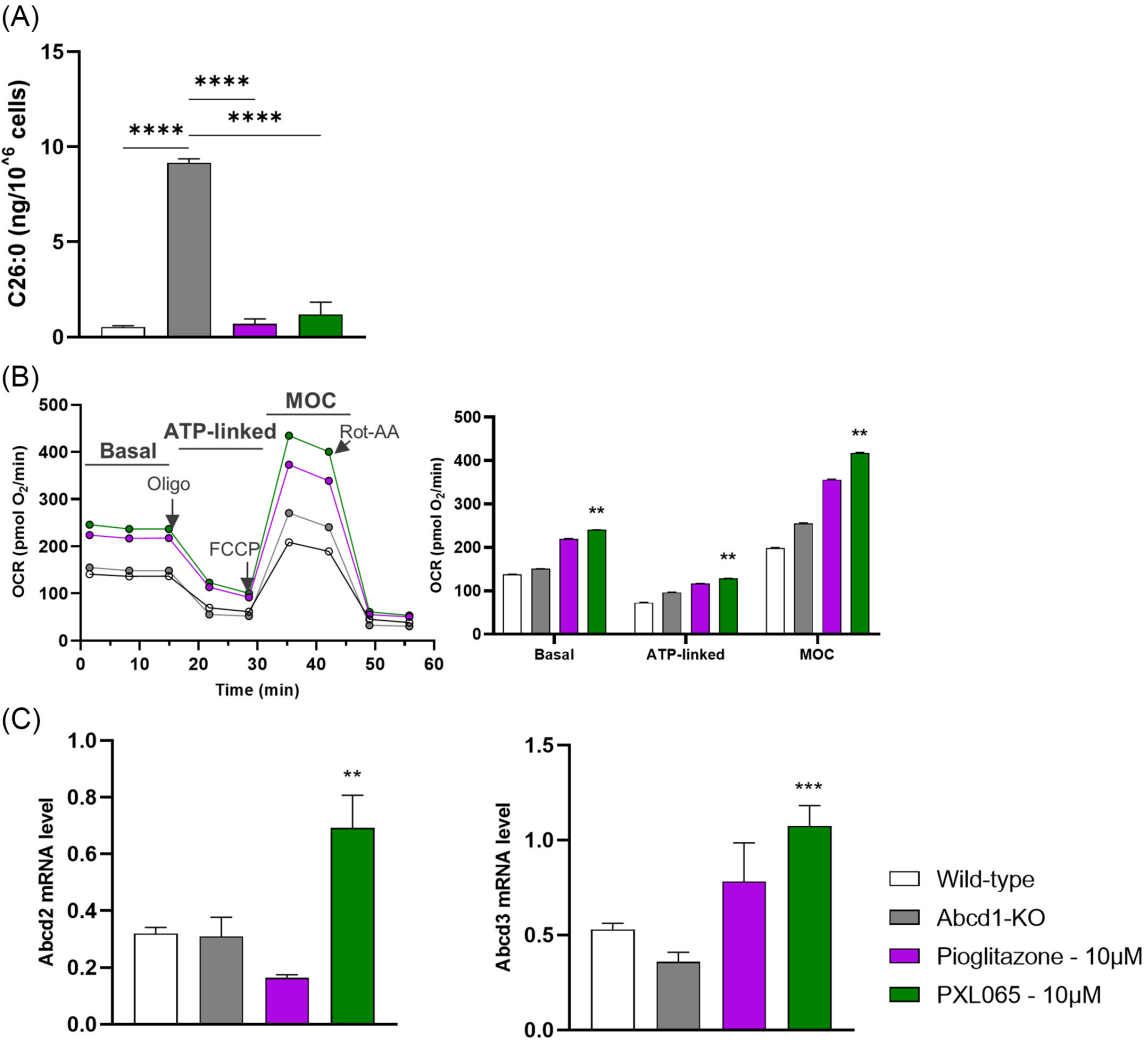
PXL065 improves adrenoleukodystrophy (ALD) pathology in glial cells from Abcd1‐null mice. Cells were exposed to 10 μM PXL065, pioglitazone, or control media. (A) Very long chain fatty acids (C26:0) quantitative analysis performed by mass spectrometry after 7 days exposure. (B) Bioenergetics analysis performed using a Seahorse Analyzer after 72‐h incubation; parameters were evaluated by sequential additions of: oligomycin (Oligo), FCCP and Rotenone‐Antimycin A. Basal is first three measurements, ATP‐linked is oxygen consumption rate (OCR) drop following oligo addition, Maximal Oxidative Capacity (MOC) is OCR following addition of FCCP. (C) Compensatory transporter gene expression measured by RT‐qPCR (normalized by RLP27 expression) after 72‐h incubation. Results are mean ± SEM, *n* = 2–6 replicates/condition. ** p < 0.01, *** p < 0.001, **** p < 0.0001 by one‐way analysis of variance followed by Dunnett's multiple comparison versus untreated glial cells

As glial cell dysfunction and apoptosis are key defects in ALD,^3^ we evaluated the effects of compounds exposure in glial cells from Abcd1‐null mice. As seen in patient‐derived cells, C26:0 levels were markedly increased in diseased versus WT cells (Figure 3A). Both PXL065 (− 92%, p < 0.0001) and pioglitazone (−87%, p < 0.0001) normalized C26:0 levels in Abcd1‐null glial cells. Although no clear mitochondrial phenotype was observed in untreated cells from Abcd1‐null mice (Figure 3B), basal and ATP‐linked OCR were increased following PXL065 treatment, by +58% (p < 0.01) and + 31% (p < 0.01), respectively, when compared to untreated cells. MOC was also increased by PXL065 treatment (+63%, p < 0.01). Although pioglitazone tended to improve bioenergetic parameters, its effects did not reach statistical significance. Additionally, Abcd2 and Abcd3 mRNA levels were increased by PXL065 treatment of Abcd1‐null glial cells but not by pioglitazone (Figure 3C).

### 3.4 Effects on inflammatory gene expression profiles

**FIGURE 4:**
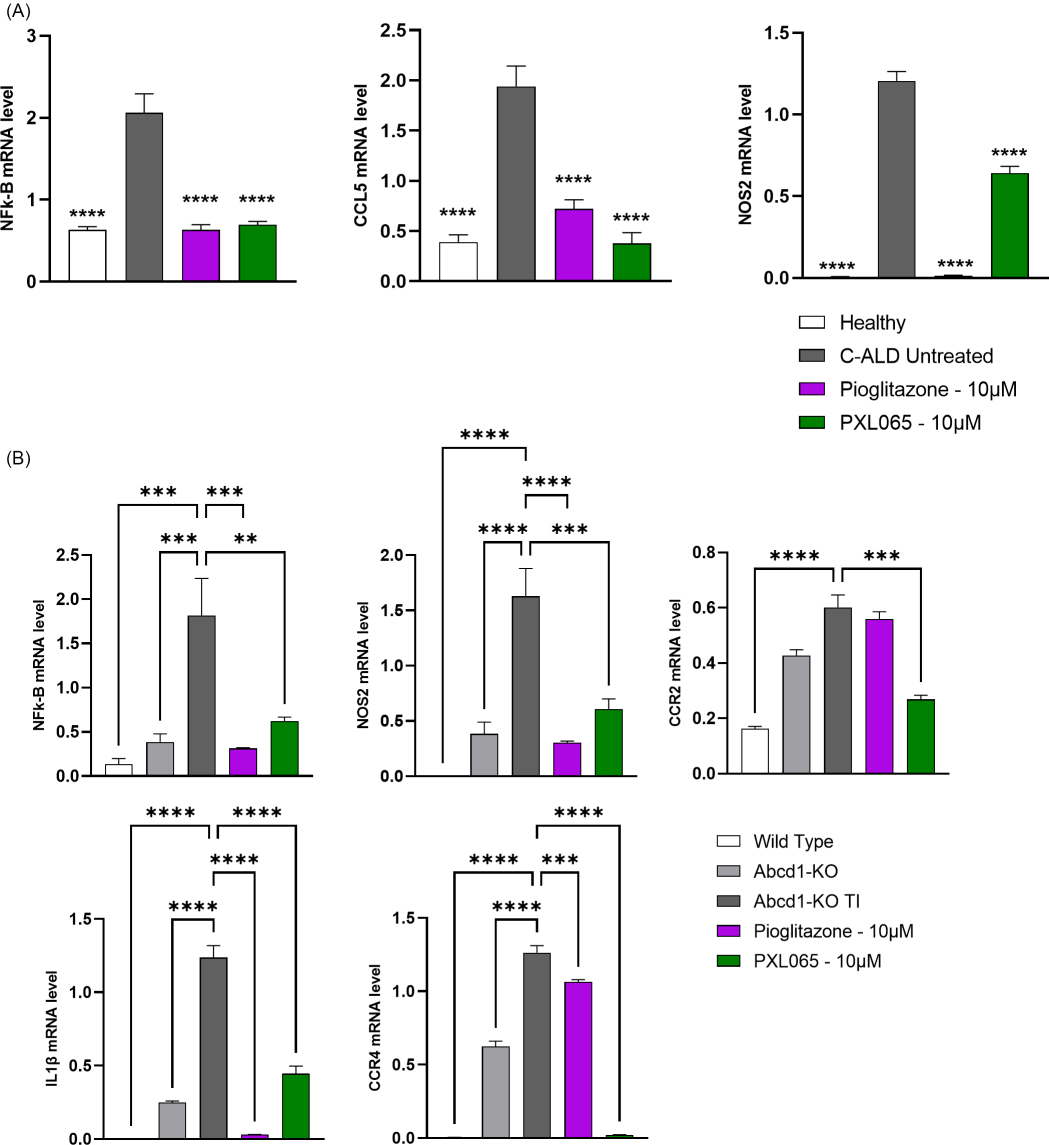
PXL065 represses pro‐inflammatory gene expression in C‐ALD patient‐derived lymphocytes and in glial cells from Abcd1‐null mice. C‐ALD lymphocytes (A) were exposed for 72 h to PXL065 or Pioglitazone at 10 μM prior to mRNA level analysis by RT‐qPCR. Abcd1 null glial cells (B) were incubated with PXL065 or Pioglitazone at 10 μM for 2 h prior to stimulation by TNFα and IL1β for 70 h (total drug exposure of 72 h), followed by mRNA level analysis by RT‐qPCR. Results are mean ± SEM, *n* = 3 replicates/condition. Results were normalized by RLP27 expression. ** p < 0.01, *** p < 0.001, **** p < 0.0001 by One‐way ANOVA followed by Dunnett's multiple comparison vs untreated C‐ALD or Abcd1 null gial cells.

In line with previous studies showing the role of inflammation in pathophysiology,^16^ C‐ALD lymphocytes exhibited increased expression of several pro‐inflammatory genes (Figure 4A). Importantly, PXL065 was able to attenuate these increases; mean mRNA level reductions in NFk‐B (−66%, p < 0.0001), CCL5 (−81%, p < 0.0001) and NOS2 (−47%, p < 0.0001) were observed. Pioglitazone exhibited similar profiles (Figure 4A).

After stimulation with TNFα and IL1β, a proinflammatory gene expression profile was also observed in Abcd1‐null glial cells versus WT mouse glial cells (Figure 4B); this pattern was significantly improved by PXL065 and pioglitazone treatment. Several mRNAs encoding inflammatory mediators were significantly repressed: NFκB (−66%, p < 0.01), NOS2 (−63%, p < 0.0001), CCR2 (−55%, p < 0.001), CCR4 (−98%, p < 0.0001), and IL1b (−64%, p < 0.0001) (Figure 4B).

### 3.5 PXL065 improves disease‐associated phenotypes in Abcd1‐null mice

**FIGURE 5:**
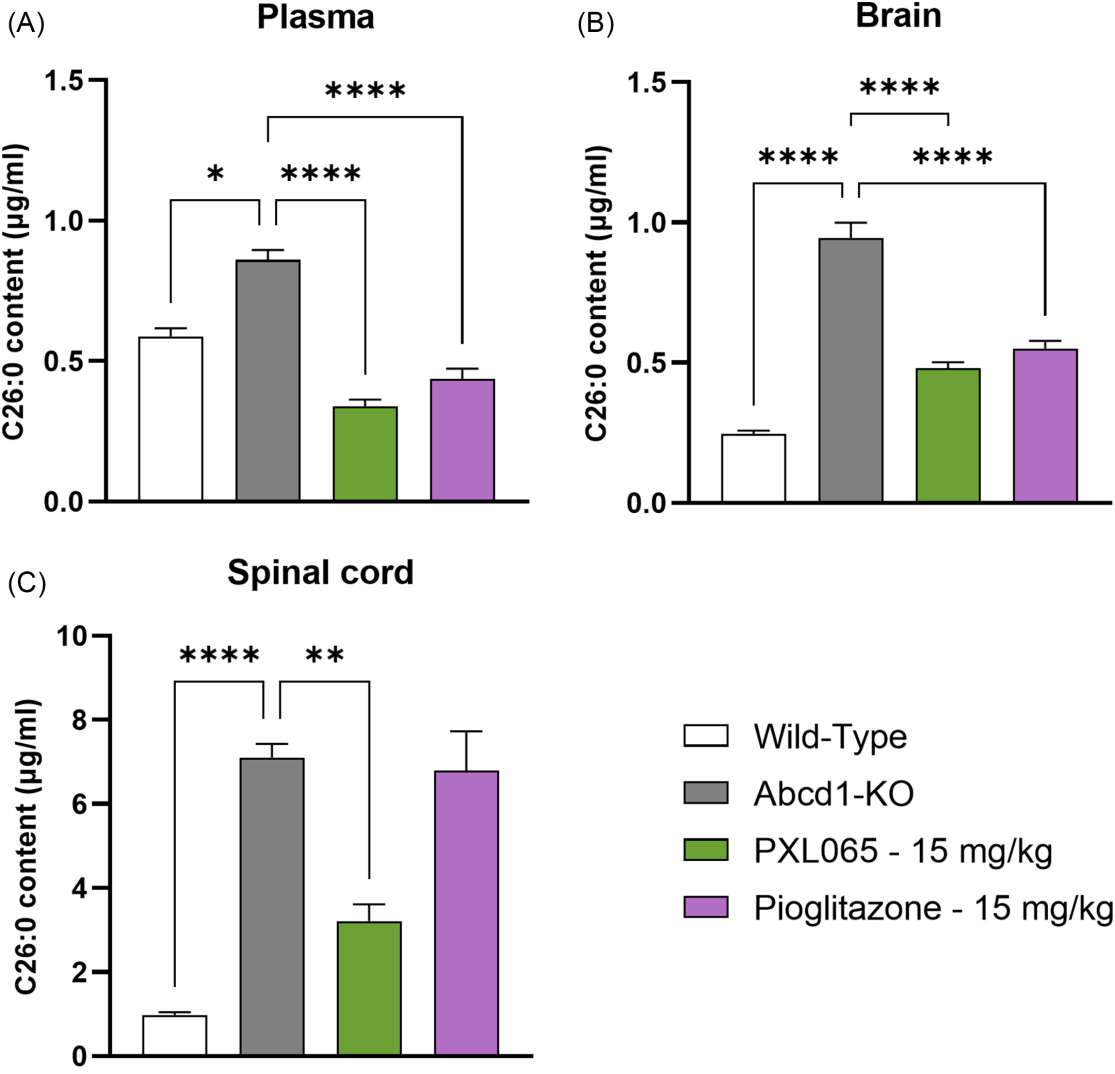
PXL065 reduces C26:0 levels in plasma, brain and spinal cord. Six‐8‐week‐old Abcd1‐null mice were treated with PXL065 or pioglitazone (15 mg/kg QD) for 8 weeks. C26:0 content was measured in plasma (A), brain (B) and spinal cord (C) by mass spectrometry. Results are mean ± SEM, n = 12 wild‐type, 15 untreated Abcd1‐null, 15 PXL065 treated Abcd1‐ null and 15 pioglitazone treated Abcd1‐null mice. * p < 0.05, ** p < 0.01, **** p < 0.0001 by one‐way analysis of variance followed by Dunnett's multiple comparison or by Kruskal–Wallis followed by Dunn's multiple comparison versus untreated Abcd1‐null mice

Abcd1‐null mice (6‐ to 8‐week‐old) were treated by oral gavage with PXL065 or pioglitazone at 15 mg/kg, once a day for 8 weeks (12 control WT, 15 untreated Abcd1 null, 15 PXL065‐treated Abcd1 null and 15 pioglitazone‐treated Abcd1‐null mice). PXL065 significantly reduced elevated C26:0 by −61% (p < 0.0001) in plasma and by −49% (p < 0.0001) in brain (Figure 5A,B) when compared to untreated Abcd1‐null mice. Pioglitazone decreased C26:0 by −48% (p < 0.0001) in plasma and by −42% (p < 0.0001) in the brain. In spinal cord, PXL065—but not pioglitazone—decreased C26:0 by −55% (p < 0.01) (Figure 5C).

**FIGURE 6:**
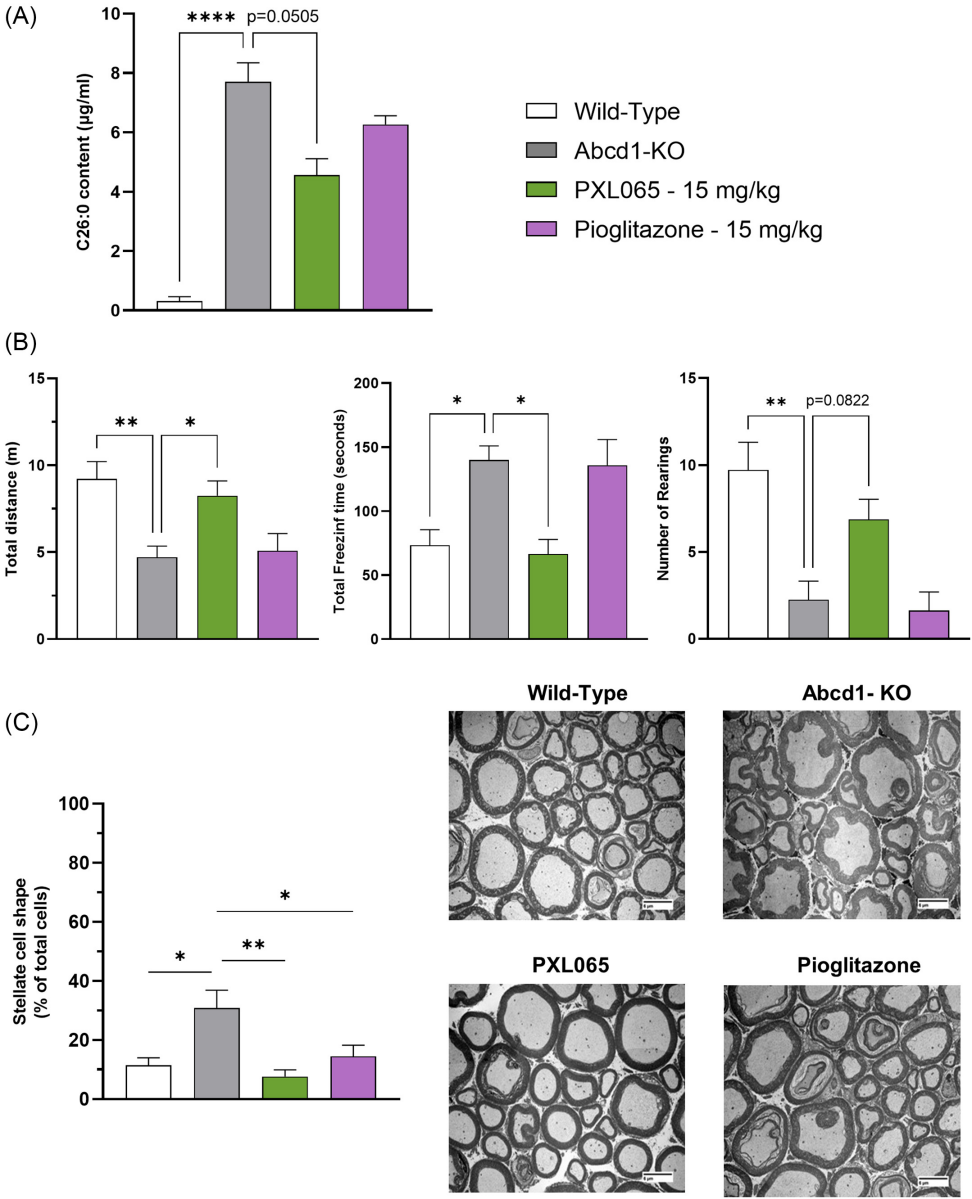
In older Abcd1‐null mice, PXL065 lowers spinal cord very long chain fatty acids, improves sciatic nerve morphology and enhances neurobehavioral functions. Older (age 13 months) Abcd1‐null mice were treated for 12 weeks with PXL065 or pioglitazone (15 mg/kg QD). (A) C26:0 content measured in spinal cord by mass spectrometry; results are mean ± SEM, *n* = 8 animals/condition. (B) Open‐field test monitoring results. Data are mean ± SEM, *n* = 8 animals/condition (seven animals for wild‐type in graph presenting number of rearings). (C) Axonal morphology (with representative images) of neurons determined by morphometric analysis of transversal slices of the sciatic nerve by electronic microscopy (800×). Data are mean ± SEM, *n* = 4 animals/condition. * p < 0.05, ** p < 0.01, **** p < 0.0001 by one‐way analysis of variance followed by Dunnett's multiple comparison or by Kruskal–Wallis followed by Dunn's multiple comparison vs untreated Abcd1‐null mice

The neurologic phenotype of Abcd1‐null mice is subtle and is generally only manifested in older mice.^17^ To interrogate the potential for in vivo efficacy in this context, 13‐month‐old Abcd1‐null mice were treated orally with PXL065 or pioglitazone at the 15 mg/kg dose level for 12 weeks (*n* = 8 animals per condition). Similar to the effects observed in younger animals, spinal cord C26:0 content was elevated in untreated mice and was improved (−41%, p = 0.0505) by chronic PXL065 treatment (Figure 6A) while pioglitazone did not show any apparent effect.

Neurobehavioral functions were assessed using open‐field test monitoring, a global assessment of spontaneous locomotor activity^18^ (eight animals per condition). At the end of the treatment period (i.e., at 16 months old), Abcd1‐null mice exhibited impaired locomotor function, shown by a decrease of mean total distance traveled and number of rearings, and an increase in mean freezing time compared to age‐matched WT mice (Figure 6B; seven animals for WT—number of rearings). Importantly, PXL065, but not pioglitazone, nearly normalized the mean total distance traveled (p < 0.05) and improved the mean freezing time (p < 0.05). Although not significant (p = 0.08), PXL065 tended to also increase the number of rearings. Altogether, these results indicate an improvement of locomotor function in the context of exploratory behavior in Abcd1‐null mice treated with PXL065.

In this long‐term study of older mice, sciatic nerve morphology was also assessed by EM (Figure 6C). An increase in the proportion of cells with an abnormal stellate shape (+31%, p < 0.05) was noted in Abcd1‐null mice compared to WT. Both PXL065 (−63%, p < 0.01) and pioglitazone (−37%, p < 0.05) significantly reduced the proportion of cells with stellate shape when compared to untreated mice.

The effects of PXL065 and pioglitazone on the different parameters measured in vitro and in vivo are summarized and compared in Table 1.

**TABLE 1:** overview of the parameters measured and comparison between PXL065 and pioglitazone effects. Green identifies similar results between PXL065 and pioglitazone, orange identifies different results

	Parameter	Model		Effect of PXL065	Effect of pioglitazone
In vitro	VLCFA	ALD cell lines	AMN fibroblasts	Decrease in C26:0 and C24:0	Decrease in C26:0 and C24:0
			C‐ALD fibroblasts	Decrease in C26:0	Decrease in C26:0
			AMN lymphocytes	Decrease in C26:0 and C24:0	Decrease in C26:0 and C24:0
			C‐ALD lymphocytes	Decrease in C26:0 and C24:0	Decrease in C26:0 and C24:0
		Abcd1 null mice glial cells		Decrease in C26:0	Decrease in C26:0
	Bioenergetics	ALD cell lines	AMN fibroblasts	Increase in basal and ATP‐linked OCR	No effect
			C‐ALD fibroblasts	Increase in basal and ATP‐linked OCR	Increase in basal OCR only
			C‐ALD lymphocytes	Increase in basal, ATP‐linked and maximal OCR	Decrease in ATP linked OCR
		Abcd1 null mice glial cells		Increase in basal, ATP‐linked and maximal OCR	No effect
	ABCD2 – ABCD3 transporters	ALD cell lines	AMN fibroblasts	Increase in ABCD2 and ABCD3 expression	Increase in ABCD2 and ABCD3 expression
			C‐ALD fibroblasts	Increase in ABCD2 and ABCD3 expression	Increase in ABCD2 expression only
			AMN lymphocytes	Increase in ABCD2 and ABCD3 expression	Increase in ABCD2 and ABCD3 expression
			C‐ALD lymphocytes	No effect	No effect
		Abcd1 null mice glial cells		Increase in ABCD2 and ABCD3 expression	No effect
	Inflammation markers	C‐ALD lymphocytes		Decrease in gene expression in multiple inflammatory markers	Decrease in gene expression in multiple inflammatory markers
		Stimulated Abcd1 null mice glial cells		Decrease in gene expression in multiple inflammatory markers	Decrease in gene expression in multiple inflammatory markers
In vivo	VLCFA	6‐8‐week‐old Abcd1‐null mice		Decrease in C26:0 in plasma, brain, and spinal cord	Decrease in C26:0 in plasma and brain
		13‐month‐old Abcd1‐null mice		Decrease in C26:0 in spinal cord	No effect
	Open field test	13‐month‐old Abcd1‐null mice		Decrease in total distance and total freezing time	No effect
	Sciatic nerve axon morphology	13‐month‐old Abcd1‐null mice		Decrease in stellate cell shape percentage	Decrease in stellate cell shape percentage

Abbreviations: ALD, adrenoleukodystrophy; AMN, adrenomyeloneuropathy; C‐ALD, cerebral‐ALD; OCR, oxygen consumption rate; VLCFA, very long chain fatty acids.

### 3.6 Additional assessment of potential mechanisms

**FIGURE 7:**
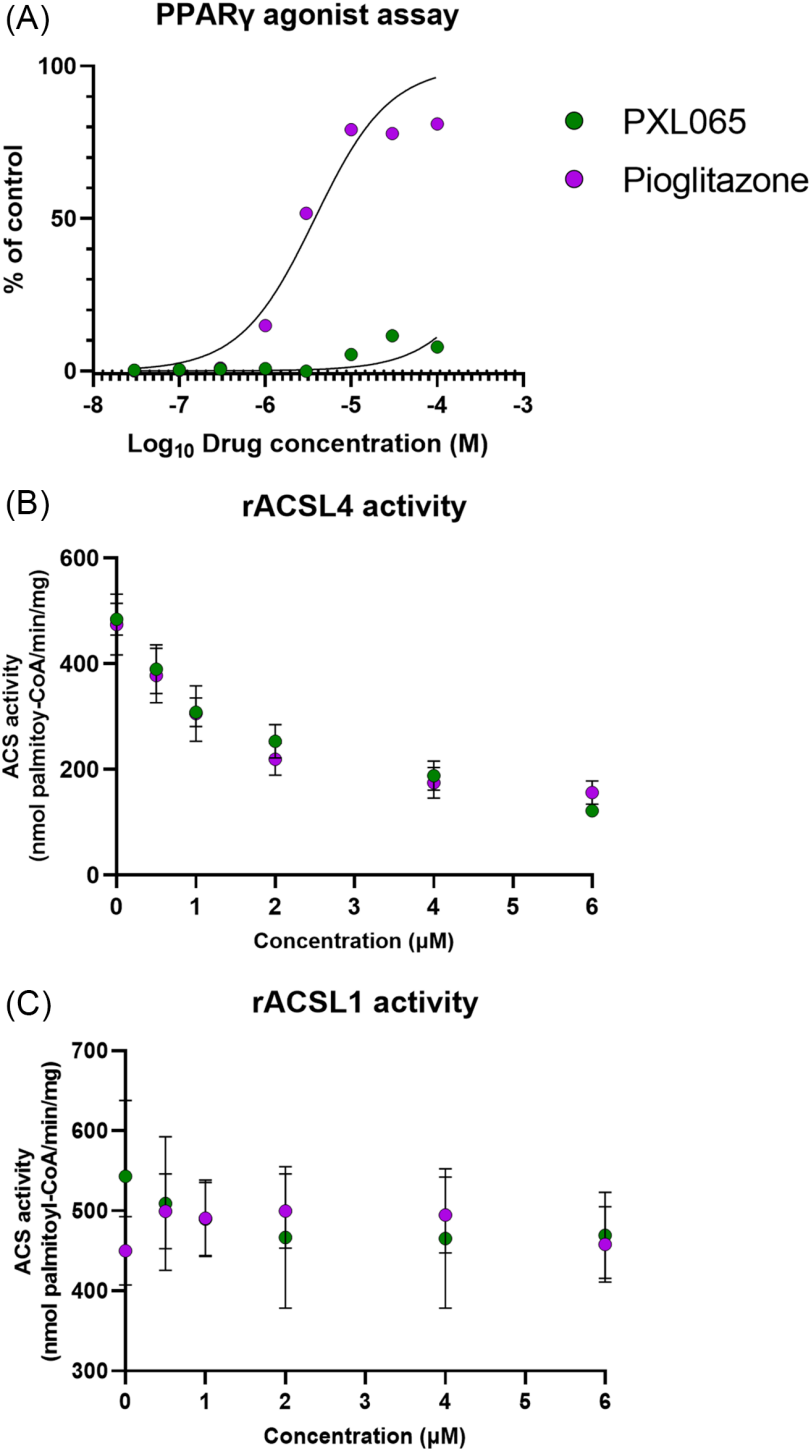
PXL065 retains nongenomic actions—inhibition of ACSL4—without PPARγ agonist activity. Enzymatic activities were measured by ex situ biochemical assays. PPARγ agonist activity (A) was measured using recombinant enzyme with a cofactor recruitment fluorescence assay using recombinant ligand binding domain protein in the presence of increasing concentrations of PXL065 or pioglitazone from 0.03 to 100 μM. Results are expressed as a percent of the control response to 10 μM rosiglitazone. ACSL4 (B) and ACSL1 activity (C) assays were performed using recombinant enzymes and [14C] palmitate, in the presence of increasing concentrations of PXL065, pioglitazone and leriglitazone from 0.5 to 6 μM. Results are mean ± SEM, *n* = 3 experiments for ACSL and 2 experiments for PPARγ.

To further interrogate the potential molecular mechanism(s) of action of PXL065, we performed ex situ biochemical assays to measure the activity of the known targets of pioglitazone. We first confirmed its relative lack of PPARγ agonism (Figure 7A). PPARγ agonist potency was markedly greater for pioglitazone (EC_50_ = 3.8 μM) than for PXL065 (EC_50_ > 100 μM). Pioglitazone and other TZDs have previously been shown to inhibit a nongenomic target—ACSL4, without any effect on the ACSL1 isoform.^19^, ^20^ In in vitro ACS assays, both pioglitazone and PXL065 inhibited recombinant rat ACSL4 activity with the same potency (IC_50_ = 2.08 and 2.12 μM, respectively) (Figure 7B). As expected, there was no inhibition of recombinant rat ACSL1 activity (Figure 7C). A visual summary of the targets of PXL065 and Pioglitazone is presented in Figure 7D.

## 4 DISCUSSION

New therapeutic approaches for the treatment of patients with ALD are urgently needed to address the unmet medical needs associated with this debilitating monogenic neurometabolic disease. Although allogeneic hematopoietic stem cell transplantation was shown to be effective to treat C‐ALD, it is a highly invasive procedure that is not without risks, such as graft failure and infections from opportunistic pathogens.^21^ Of note, the procedure yields more favorable outcomes in the context of early‐stage disease.^21^

PXL065 is a novel TZD and the deuterium‐stabilized (*R*)‐enantiomer of pioglitazone that is currently in Phase 2 clinical development for non‐alcoholic steatohepatitis (NASH, ClinTrials.Gov ID:NCT04321343). Since pioglitazone has been reported to ameliorate features of ALD in preclinical models,^9^ we sought to test the hypothesis that PXL065 could also be an attractive therapeutic candidate for ALD.

In a series of in vitro experiments in two ALD cells lines derived from one AMN and one C‐ALD patient, PXL065 improved multiple disease‐related phenotypes. Remarkably, PXL065 nearly normalized elevated C26:0 content. These effects were accompanied by a reduction of elevated C24:0 levels without changing the levels of C22:0, suggesting specificity to the VLCFA species that are associated with ALD.^2^ Of note, the molecular mechanisms linking PXL065 to the VLCFA lowering we observed are yet to be determined. Further experiments would be required to decipher whether suppression of very long chain fatty acid elongase and/or conversion to monounsaturated forms and/or improved β‐oxidation are at play.

Mitochondrial dysfunction is described as a key component of the pathophysiology, which could contribute to the clinical onset through a decrease in β‐oxidation activity.^22^ VLCFA‐induced cytotoxicity is also reported to target cellular energy‐dependent functions.^23^ In addition, ultrastructural examination of neural tissue from AMN patients also reveals abnormal mitochondria.^24^ Increased ATP‐linked OCR mediated by PXL065 could contribute to preserving a broader range of cellular energy‐dependent functions altered in the disease. Although VLCFA catabolism is mostly mediated by peroxisomes, we speculate that increased OCR in response to PXL065 might enhance mitochondrial lipid oxidation and contribute to VLCFA lowering by driving catabolism of shorter chain length fatty acid precursors that are substrates for the elongase that catalyzes synthesis of C24:0 and C26:0.^25^ Overall, an increase in mitochondrial efficiency mediated by PXL065 could support essential cellular functions and participate in global phenotypic improvements.

Although somewhat controversial,^26^ current evidence indicates that ABCD2 (and ABCD3) have VLCFA transport capacity and could compensate for the absence of ABCD1.^15^, ^27^ Thus, overexpression of ABCD2 has been proposed as a therapeutic approach for ALD.^28^, ^29^ The increased expression of ABCD2 induced by PXL065 might, therefore, contribute to lower VLCFA levels found in patient‐derived cells. Although we did not assess this hypothesis in vivo, ex vivo data obtained in glial cells from Abcd1‐null mice provide further support for this potential mechanism.

Cerebral ALD is characterized by an important inflammatory component.^30^ The transition to overt C‐ALD is not well understood but might involve low‐grade inflammation with environmental triggers leading to uncontrolled inflammation. Of interest, TZD's (including pioglitazone) were reported to attenuate cytokine production by human immune cells^31^ and to protect from neuroinflammation via effects on peroxisomes.^32^ Accordingly, our results showed that PXL065 has the potential to repress pro‐inflammatory gene expression in Abcd1‐null mouse‐derived glial cells and C‐ALD patient‐derived lymphocytes. Although we did not assess this parameter in vivo, PXL065 was previously shown to attenuate inflammation in vivo in the *db/db* mouse model of metabolic syndrome.^11^

The most widely used in vivo model of ALD is the Abcd1‐null mouse. This model exhibits a phenotype consistent with AMN, including increased VLCFA levels in plasma and tissues beginning at an early age^33^ as well as the later development of neurological impairment.^17^ In this context, PXL065 reduced elevated VLCFA in plasma, spinal cord and brain. In addition, PXL065 ameliorated impaired locomotor function in 13‐month‐old mice, suggesting an improvement of the progression of neurologic dysfunction. This effect coincided with reduced VLCFA in spinal cord but may also have resulted from additional positive effects on mitochondrial function, as demonstrated ex vivo, for instance. Importantly, PXL065 treatment improved the proportion of abnormally shaped axons in sciatic nerve from Abcd1‐null mice. This feature has not been previously reported in mice. However, there are reports of similar findings in peripheral nerve and dorsal root ganglia from patients with AMN including swelling of individual axons, lamellar changes in myelin and segmental demyelination.^34^ On balance, the in vivo efficacy achieved with PXL065 in two independent long‐term studies in Abcd1‐null mice further supports the pursuit of this compound as a possible therapy for ALD.

The beneficial effects observed with PXL065 appeared to be superior to pioglitazone for several parameters at the same concentrations and doses (see comparison in Table 1). Differences included: improvements in mitochondrial function in patient‐derived and mouse glial cells, Abcd2 and Abcd3 mRNA induction in diseased glial cells, reductions in spinal cord VLCFA levels, and effects on all three main parameters measured in the open‐field test. In the more severe Abcd1/Abcd2 double knockout model, Morato et al.^9^ reported that pioglitazone improved axonal degeneration and locomotor deficits without significantly impacting VLCFA levels. Reasons for the latter discrepancy are uncertain although a lower (9 mg/kg/day) dose of pioglitazone was employed and administered in the diet instead of by oral gavage. Leriglitazone is a related TZD, also derived from pioglitazone, which is in clinical development in ALD patients (ClinTrials.gov ID: NCT03231878). In both Abcd1‐null and Abcd1/Abcd2 double knockout mice, leriglitazone exerted efficacy including evidence of reduced oxidative stress, improved mitochondrial function and improved motor function.^10^ Of interest, leriglitazone was not reported to significantly affect plasma VLCFA; in vivo.^10^

**FIGURE 8:**
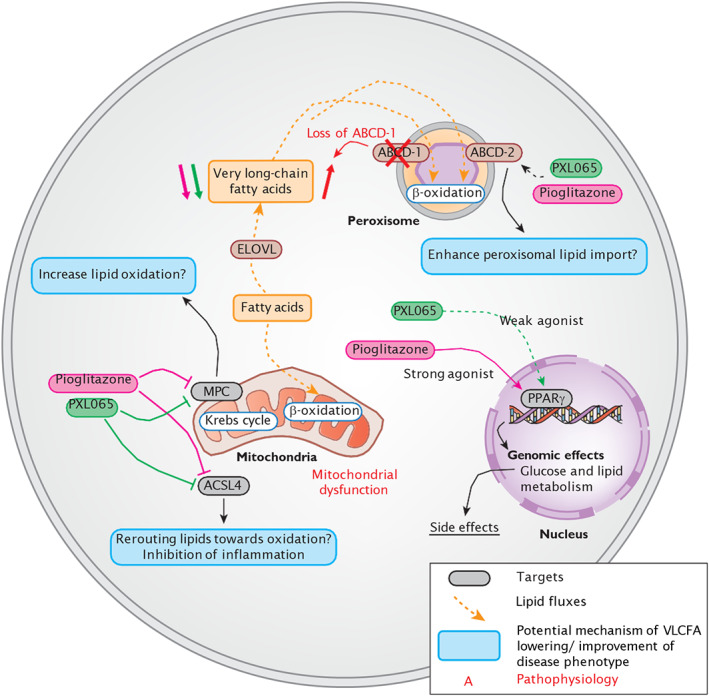
Diagram depicting the effects of PXL065 and Pioglitazone on PPARγ, the mitochondrial pyruvate carrier (MPC) and ACSL4, and the associated pathways. Potential mechanisms linking the targets of the compounds of interest to very long chain fatty acids lowering and their efficacy on the disease phenotype are represented in the blue tags.

As illustrated in Figure 8, unlike pioglitazone or leriglitazone, PXL065 has minimal intrinsic PPARγ agonist activity. However, molecular mechanisms underlying its observed beneficial effects in ALD models may still include residual PPARγ action since partial conversion of (*R*)‐ (PPARγ inactive) to (*S*)‐(PPARγ active) pioglitazone occurs between dosing intervals in cell culture and in vivo.^11^ Nevertheless, after repeated administration, steady‐state ratios of R:S enantiomers achieved with PXL065 are substantially (four‐ to eight‐fold) greater than those seen with pioglitazone—including in human subjects.^11^ In addition, we previously demonstrated a lack of PPARγ‐mediated effects in vivo in mice (weight gain, fluid retention, or increases in adiponectin) at the same dose level of PXL065 (15 mg/kg) that was used in Abcd1‐null mice here.^11^ Therefore, we envision that the efficacious dose levels of PXL065 could potentially be achieved in patients with ALD without the known liabilities of weight gain, edema, and bone loss that are mediated by stronger PPARγ agonism (as illustrated in Figure 8).

Apart from PPARγ, TZDs (including pioglitazone) modulate two distinct nongenomic pathways (as illustrated in Figure 8). We previously demonstrated that PXL065 inhibits MPC with a potency equivalent to pioglitazone—IC50's 7–9 μM,^11^ and leriglitazone also inhibits MPC with an IC_50_ of 4.1 μM.^35^ Selective inhibition or genetic deletion of MPC is known to reduce intracellular lipid accumulation and inflammation in fatty liver disease models.^36^, ^37^ Such effects could potentially also be relevant in the context of ALD pathophysiology. Of greater potential importance, a specific MPC inhibitor was also shown to exert efficacy in models of neurodegeneration—specifically, to reduce neuroinflammation and improve locomotor function in Parkinson's disease models.^38^

In the present study, we also determined that, like pioglitazone,^20^ PXL065 serves as a selective in vitro inhibitor of purified ACSL4 (vs. ACSL1) activity (as illustrated in Figure 8). ACSL4 is expressed in multiple tissues including brain and adrenal, which are affected in ALD.^39^ ACSL4's preferred substrates include unsaturated lipids (PUFA, HUFA) lipids (e.g. arachidonic acid),^40^ which are distinct from saturated VLCFA (e.g. C26:0). However, ACSL4 is implicated broadly in lipid channeling in cells.^41^ For example in an in vivo model, ACSL4 activity was shown to be important in regulating the incorporation of arachidonic acid into phospholipids as well as the downstream effects of diet‐induced obesity, including adipose tissue inflammation.^41^ Most importantly, ACSL4 is now well established as a major driver of lipid peroxidation‐mediated and iron‐dependent cell death, or ferroptosis.^42^, ^43^ TZD‐mediated inhibition of ferroptosis was shown to be tissue‐protective in the setting of pulmonary ischemia.^44^ Moreover, knockdown of ACSL4 protected mice against brain ischemia and neuroinflammation, whereas, forced overexpression of ACSL4 reportedly exacerbated ischemic brain injury.^45^ Ferroptosis is now being increasingly considered as a component of several neurodegenerative diseases.^43^, ^46^ Although no link between ALD pathophysiology and ferroptosis or ACSL4 has been established to‐date, we speculate that inhibition of ACSL4 might also contribute to long‐term beneficial effects of PXL065 (or pioglitazone) to attenuate neuroinflammation or neuronal cell loss in the context of ALD. Future studies will be required to further assess if or how inhibition of either MPC or ACSL4—versus residual PPARγ agonist activity—could impact cellular VLCFA accumulation or downstream features of ALD pathophysiology.

In the present series of experiments, we characterized PXL065 in well‐accepted preclinical models of ALD and demonstrated that it has the potential to suppress VLCFA accumulation, the proximate driver of disease, and to also attenuate other features of disease pathophysiology. The in vivo efficacy profile of PXL065 also suggests that clinical development could proceed by first assessing effects on biomarkers of disease followed by long‐term trials designed to confirm clinical benefits. A clinical development program that is initially focused on adult patients with AMN is planned to begin in 2022.

## AUTHOR CONTRIBUTIONS

**Pierre‐Axel Monternier**, **Jaspreet Singh**, **Eric Klett**, **David E. Moller**, and **Sophie Hallakou‐Bozec** designed the experiments, analyzed results, and participated to the writing of the manuscript. **Parveen Parasar**, **Navtej Kaur**, and **Tavarekere N. Nagaraja** performed the experiments. **Pierre Theurey**, **Sheila DeWitt**, and **Vincent Jacques** participated to the writing of the manuscript.

## FUNDING

This work was supported by funds from Poxel. Jaspreet Singh is also supported by National Institute of Health grants NS114245 and NS114775. Navtej Kaur is supported by funds from Henry Ford Health System.

## CONFLICT OF INTEREST

Pierre‐Axel Monternier is an employee and shareholder of Poxel. Jaspreet Singh received sponsored research grants from Poxel to support the conduct of experiments related to the aims of this manuscript and is also a member of Poxel's Scientific Advisory Board. Parveen Parasar declares no conflict of interest. Pierre Theurey is an employee and shareholder of Poxel. Sheila DeWitt received sponsored research grants from Poxel to support the conduct of experiments related to the aims of this manuscript. Vincent Jacques received sponsored research grants from Poxel to support the conduct of experiments related to the aims of this manuscript. Eric Klett received sponsored research grants from Poxel to support the conduct of experiments related to the aims of this manuscript. Navtej Kaur declares no conflict of interest. Tavarekere N. Nagaraja declares no conflict of interest. David E. Moller is an employee and shareholder of Poxel. Sophie Hallakou‐Bozec is an employee and shareholder of Poxel.

## Supporting information


**Appendix S1** Supporting information

## Data Availability

The data that support the findings of this study are available from the corresponding author upon reasonable request.
