# Supplementary material for: Therapeutic potential of deuterium‐stabilized (R)‐pioglitazone—PXL065—for X‐linked adrenoleukodystrophy
Source: J Inherit Metab Dis. 2022 May 19;45(4):832–47. doi: 10.1002/jimd.12510 (PMC9545763; doi:10.1002/jimd.12510)
Supplement: Supplementary file 1 — Appendix S1 Supporting information [file JIMD-45-832-s001.docx]

**Therapeutic potential of deuterium-stabilized (*R*)-pioglitazone - PXL065 - for X-linked adrenoleukodystrophy**

**Supplementary Materials and Methods**

VLCFA content

*VLCFA Analysis*

Samples are adjusted to a final volume of 0.5 – 1 mlL with LC-MS grade water and spiked with 10 ng of lignoceric acid-d4 as an internal standard. The sample is acidified to pH 3-4 with dilute hydrochloric acid and extracted with isooctane-ethyl acetate (9:1) three times with equal volume. The extract is dried under nitrogen and the residue is reconstituted in methanol-water-ammonium acetate (75:25: 10 mM).

*Total fatty acids*

After preparing the samples with internal standard as described above for free fatty acids, aqueous sodium hydroxide is added to a final concentration of 1 M. The mixture is incubated at 37 ˚C in dark under nitrogen for 3hrs. The samples are then acidified, extracted, and reconstituted as described above.

*LC-MS analysis of fatty acids*

The reconstituted fatty acid extracts are subjected to HPLC on Targa C8 column (2x10 mm) using methanol-aqueous ammonium acetate (10 mM) solvent mixture. The column is eluted with a gradient of methanol (75 to 90%) over 8 min at a flow rate of 0.25 ml/min. The column eluent is directly introduced to mass analyzer (QTRAP5500) and monitored for fatty acids using published pseudo MRM method^1^. Under these conditions, the VLCFA elute between 5 and 8 min. Each fatty acid is quantitated against the added internal standard.

ACS activity

Purified rat FLAG-ACSL1 and FLAG-ACSL4 were produced as previously described (PMID: 28209804). The ACS activity assay reaction contained 50mM [14C] palmitate in Triton X-100, 1mM EDTA, 10 mM ATP, 250 mM CoA, 175 mM Tris (pH 7.5), 8 mM MgCl_2_, and 5 mM dithiothreitol in a total reaction volume of 200 µL. The maximum concentration of Triton X-100 in the assay was 0.03% (0.5 mM). The reaction was initiated with 1 µg of purified protein for FLAG-rACSL1 or FLAG-rACSL4. ACS activity was measured after a 10-min incubation at room temperature. Substrate concentrations and reaction time allowed for measures of initial rates. Each reaction was performed in duplicate for each condition. Purified recombinant rat FLAG-ACSL1 and FLAG-ACSL4 were assayed in the presence of TZD compounds pioglitazone and PXL065 at 0, 1, 1.5, 2, 4, and 6 µM final concentration. Reactions were carried out in 13x100 mm glass test tubes. Following 10 min reaction time, the reaction was terminated with Dole’s solution. Organic and aqueous phases were separated, and the aqueous phase was isolated. 600 µL of the aqueous phase (containing radiolabeled palmitoyl-CoA) was mixed with 4 mL of Eco-lite and counted in a Wallac 1400 scintillation counter using Easy Count using C14_DPM for calculations. One experiment was considered a single ACSL isoform with a single compound at any given concentrations. Three experiments with reactions in duplicate were conducted for each treatment.

PPARγ agonist assay

PPARγ agonist activity was measured using Histidine-tagged-PPARγ (Ligand Binding Domain). 50 nM proteins are mixed with 50 nM biotin-tagged-TRAP220 coactivator and 0.4 µg fluorescence acceptor (anti-histidine antibody coupled-beads) in an incubation buffer containing 20 mM Hepes/NaOH (pH 7.4), 80 mM Nacl, 0.08% Tween 20 and 0.8 mM DTT and 0.08% BSA. The mixture is pre-incubated for 30 min at 22°C in the presence of one of the following: incubation buffer (basal control), the reference agonist at 10 µM (rosiglitazone, stimulated control) or various concentrations (EC_50_ determination) of PXL065 or pioglitazone. Thereafter, fluorescence donor (streptavidin coupled-beads) is added at a final concentration of 0.4 µg. Following 120 min incubation at 22°C, the signal is measured at λex=680 nm and λem=520 and 620 nm using a microplate reader (EnVision, Perkin Elmer).

Animals

C57BL/6J (Stock N°000664) mouse breeding pairs and Abcd1 null (B6.129-Abcd1^tm1Kan^/J, Stock No: 003716) breeding pairs were purchased from Jackson Laboratory (Bar Harbor, ME) and maintained at the Henry Ford Health System (HFHS) animal facility on a 12/12hrs light/dark cycle and provided standard rodent chow and water *ad libitum*. All animal procedures were approved by the HFHS Animal Review Committee (IACUC#1050), and all animals received human care in compliance with the HFHS experimental guidelines and the National Research Council’s criteria for humane care (Guide for Care and Use of Laboratory Animals).

Genotyping was performed by PCR on tail-clip samples using DirectPCR (Tail) Lysis Reagent for Genotyping (Viagen, #102-T) and FailSafeTM PCR2X Premix E (Lucigen, #FSP995E). Primers used : mALD Common: 5’-CACAGCCTCTCTCCTTAAGACC-3’ ; mALD-WT: 5’-CTCGTTGTCTAGGCAACTGG-3’ ; mALD-Mutant: 5’-CTTCTATCGCCTTCTTGACG-3’.

Open Field Test

All behaviors were recorded in an undisturbed environment, in the mornings at the same time of the day, for an hour a day, for 4 consecutive days to reduce variability in the behavioral measurements. Individual mice were placed in the center of a transparent plexiglass open field maze (W x D x H; 260 x 260 x 400 mm) and behavioral activity was recorded for latter analysis with ANYmaze software using a camcorder positioned above the apparatus. The chamber was cleaned between each mouse using 70% Ethanol and permitted to dry.

Axonal Morphology

Briefly, sciatic nerve samples were harvested from glutaraldehyde-perfused mice and fixed in 25% glutaraldehyde for 24 hours, rinsed in phosphate buffered saline (PBS) for 15 min followed by post-fixation in a 1:1 ratio of 1% osmium tetroxide and PBS for 2hrs. The sample was washed in distilled water 2x's for 15 min and dehydrated in 50%, 75%, and 95% alcohol and finally in propylene oxide. The sample was then infiltrated with a 1:1 mixture of propylene oxide and araldite resin and placed in a vacuum with 100% araldite resin overnight. Infiltrated samples were embedded in the proper mold with araldite resin and cured overnight at 60° C. Approximately 120 nm thin sections were cut using a Leica EM UC7 ultratome and collected on 200 mesh copper grids. The grids were allowed to dry and stained with uranyl acetate for 5 min (rinsed) and lead citrate for 5 min (rinsed). Grids were visualized using a JEOL 1400 Flash TEM and imaged with a BioSprint camera and AMT image capture software.

References

1. Hellmuth, C., Weber, M., Koletzko, B. & Peissner, W. Nonesterified fatty acid determination for functional lipidomics: comprehensive ultrahigh performance liquid chromatography-tandem mass spectrometry quantitation, qualification, and parameter prediction. *Anal Chem.* 2012;84(3):1483-1490

**Supplementary Figures**

AMN/C-ALD Untreated

**Supplementary Figure 1: PXL065 and pioglitazone improve VLCFA levels in lymphocytes derived from two ALD – C-ALD and AMN** **– patients.** Effect of PXL065 and Pioglitazone on VLCFA levels measured by mass spectrometry in AMN and C-ALD lymphocytes, following incubation with the drugs at 10 µM for 7 days. Results are mean ±SEM, n=3 replicates / condition / patient. ** p<0.01, *** p<0.001, **** p<0.0001 by One-way ANOVA followed by Dunnett’s multiple comparison vs untreated AMN/C-ALD cells.

**Basal**

**ATP-linked**

**MOC**

Oligo

FCCP

Rot-AA

C-ALD Untreated

**Supplementary Figure 2: PXL065 improves mitochondrial function in patient-derived lymphocytes.** C-ALD lymphocytes were exposed for 72hrs to PXL065 or Pioglitazone at 10µM. Bioenergetics analysis were performed using a Seahorse Analyzer and parameters were evaluated by sequential additions of: oligomycin (Oligo - 1 μM), FCCP (0.25 μM) and Rotenone-Antimycin A (Rot-AA - 1 μM). Basal is first three measurements, ATP-linked is OCR drop following oligo addition, Maximal Oxidative Capacity (MOC) is OCR following addition of FCCP. Results are mean ±SEM, n=6 replicates / condition. * p<0.05, ** p<0.01, *** p<0.001, **** p<0.0001 by One-way ANOVA followed by Dunnett’s multiple comparison *vs* untreated C-ALD cells.

AMN/C-ALD Untreated

**Supplementary Figure 3: PXL065 and Pioglitazone increase compensatory transporters Abcd2 and Abcd3 mRNA levels in AMN lymphocytes.** Cells were exposed for 72hrs to PXL065 or Pioglitazone at 10µM prior to mRNA levels analysis by RT-qPCR in AMN lymphocytes. Results are mean ±SEM, n= 3 replicates / condition / patient. * p<0.05, ** p<0.01, *** p<0.001 by One-way ANOVA followed by Dunnett’s multiple comparison or by Kruskal-Wallis followed by Dunn’s multiple comparison *vs* untreated AMN/C-ALD cells.
